# Acknowledgment of Reviewers 2025

**DOI:** 10.1177/29941520251412020

**Published:** 2026-05-19

Critical evaluations of articles by expert reviewers make a vital contribution to ensuring the high quality of a journal’s content. The editorial leadership of *Journal of Medical Extended Reality* is very grateful for the support of the highly qualified peer reviewers who have dedicated their time and effort to reviewing our articles. We would like to show our appreciation by thanking the following individuals for their assistance with the review of articles for the journal in 2025.*

*Data as of November 22, 2025.

Jonathan Abbas

Rahaf Al Assil

Sarah Anderson

Mohsen Annabestani

Megan Armstrong

Agnes Bakk

Ryan Beams

Alexandra Berman

Raegan Bishop

M. Bourass Bourass

Mary Lee Carter

Todd Chang

Ashish Chogle

Linda Ciavarelli

Itai Danovitch

Ja-Nae Duane

Renée El-Gabalawy

Anthony Faiola

Samir Ghandour

Jeffrey Gold

Bronwyn Griffin

Joze Guna

Ryan Harari

Nancy Haug

Olivia Henry

Ibrahim Hussain

Oranicha Jumreornvong

Oranicha Jumreornvong

Richard Kim

Monika Kornacka

Min Lang

Eva Lendaro

Omer Liran

Phil Lopes

John Luna

Jacob Lurie

Todd Maddox

Edris Mahtab

Asher Marks

Samuel Max

Sheera Minkowitz

Deepika Mohan

John Moon

Joe Morgan

Triton Ong

Margot Paul

Sameer Peesapati

Les Posen

Kavya Reddy

Angelica Rego

Albert (Skip) Rizzo

Qing Ruan

John Rubin

Korak Sarkar

Akshay Shanker

Alexandros Sigaras

Elliott Silverman

Brennan Spiegel

Raul Uppot

Douglas White

Robert White

Keith Widmeier

Hannah Williams

Andrea Won

Henry Xiang

Haipeng Zhang

